# Switching from serotonin reuptake inhibitors to agomelatine in patients with refractory obsessive-compulsive disorder: a 3 month follow-up case series

**DOI:** 10.1186/1744-859X-10-5

**Published:** 2011-02-28

**Authors:** Michele Fornaro

**Affiliations:** 1 University of Genova, Department of Psychiatry, Genoa, Italy

## Abstract

**Background:**

Serotonin reuptake inhibitors (SRIs) currently represent the cornerstone of obsessive-compulsive disorder (OCD) pharmacotherapy. However, OCD is characterized by high rates of partial and/or absent response to standard, recommended treatments, often prompting pharmacological and non-pharmacological augmentation or switching of strategies. Agomelatine, a novel melatonin agonist and selective serotonin antagonist (MASSA) antidepressant approved for major depressive disorder (MDD) has recently been additionally proposed as a treatment for anxiety disorders such as social anxiety disorder (SAD) and panic disorder (PD), but not yet OCD. Nonetheless, agomelatine may have a role in the management of OCD, essentially due to its anxiolytic 5-hydroxytryptamine (HT)2C blockade action, while melatonin (MT)1 and MT2 modulation might contribute to circadian rhythm restoration if impaired.

**Methods:**

This case series reports the outcome of six patients with or without comorbid mood and/or other anxiety disorders who were treated with SRIs at adequate doses for at least 8 weeks, showing partial or no response. Patients were then switched to agomelatine 50 mg/day, and followed up for 12 weeks.

**Results:**

Three out of six patients, in particular those with relevant circadian rhythm subjective impairment, showed a Yale-Brown Obsessive Compulsive Scale (Y-BOCS) score reduction of ≥35%. No relevant side effects were observed, but initial, transient, self-remitting dizziness in one patient and weight gain in another were seen.

**Conclusions:**

Although clinical confounding factors (subthreshold bipolarity and eventually the presence of impaired circadian rhythms) and methodological boundaries (lack of control and neurophysiological recording, tiny sample size and short follow-up) limit the validity of this preliminary observation, it does indicate agomelatine may have a role in some SRI-refractory OCD cases, thus prompting the validity of investigation by further controlled studies, even for drug-naïve OCD patients.

## Introduction

Obsessive-compulsive disorder (OCD) is a common condition that affects individuals of all ages. This disorder has been listed as one of the 10 most disabling illnesses by the World Health Organization [1], while the National Comorbidity Survey Replication indicated that OCD is the anxiety disorder with the highest percentage (50.6%) of serious cases [2]. Approximately 2% to 3% of the world's population will suffer from OCD at some point in their lives [3], and it has been estimated that most individuals with OCD spend an average of 17 years before receiving an appropriate diagnosis and treatment for their illness [4]. Additionally, OCD usually exhibits a waxing and waning course, frequently increasing in severity when left untreated, which causes unnecessary pain to those afflicted and to their families. Hence, the need for appropriate management is imperative.

Although serotonin reuptake inhibitors (SRIs), including the widely prescribed selective SRIs (SSRIs), are considered the cornerstone of pharmacological treatment of OCD [5], at least 40% of cases do not respond satisfactorily to these medications [6]. Augmentation strategies with antipsychotic medications, psychotherapies such as cognitive behavioral therapy (CBT) and others [7], as well as switching to newer classes of drugs such as the selective serotonin norepinephrine reuptake inhibitors (SNRIs) [8] have all been considered. Indeed, OCD management remains a debated issue essentially due to a still not fully understood etiopathology and to a number of eventually concomitant clinical features, mainly referable to bipolarity [9], which may account for some of the treatment-refractory cases.

Within the past few years, greater attention has been paid toward the neurobiological factors underpinning OCD, including the investigation of circadian rhythms [10] and neurosteroid [11] imbalance. Among other factors, a delayed slow wave sleep (SWS) phase [10] as well increased nocturnal secretion of adrenocorticotropic hormone (ACTH) and cortisol, a melatonin-related [12] 'stress hormone' [13], have been documented in the course of some OCD cases, although the investigation of the circadian pattern by proxy measurement (axillary temperature) essentially showed the absence of a relationship between OCD and melatonin [14], so it remains unclear if and how circadian rhythm impairment might subjectively or objectively impact on OCD burden and its perception.

Recently, the availability of agomelatine, a novel antidepressant acting as a melatonin agonist and selective serotonin antagonist (MASSA; acting against melatonin 1 (MT1) and MT2, and 5-hydroxytryptamine (HT)2C, respectively) [15] led to the exploration of its potential role even for anxiety disorders [16,17], suggesting its possible use for OCD.

This paper reports a case series of six patients with SRI-refractory OCD diagnosed with the Structured Clinical Interview for the Diagnostic and Statistical Manual of Mental Disorders, fourth edition (DSM-IV) Axis-I (SCID-I) [18] who were switched from SRIs to agomelatine and followed-up for at least 3 months. This is the first report of its kind in the literature to date.

Patients were considered refractory to an adequate treatment (administration of an effective daily dose of SRI, that is, fluoxetine 40-60 mg/day or clomipramine 225-300 mg/day, for a minimum of 8 weeks) [19] on the basis of a Yale-Brown Obsessive Compulsive Scale (Y-BOCS) score >18 [20]. All patients started agomelatine treatment at 50 mg/day (the current approved dose range for treating major depressive disorder (MDD) is 25-50 mg/day), which lasted for a minimum of 12 weeks with monthly evaluations of obsessive-compulsive (OC) symptoms through Y-BOCS administration. Administration of the Hamilton Scale for Depression (HAM-D) [21] and Hamilton Scale for Anxiety (HAM-A) [22] was performed at baseline and at week 12. The Biological Rhythms Interview of Assessment in Neuropsychiatry (BRIAN), a 18-item screening tool designed to help clinicians better assess their patients and researchers improve the evaluation of the impact of novel therapies targeting biological rhythm pathways [23], was administered at baseline and at week 12 (end point). Remarkably, considering the clinical setting of this preliminary investigation, the evaluation of circadian rhythm functioning was subjective and did not include any objective neurophysiological monitoring such as polysomnography despite the acknowledgement of reduction of sleep onset rapid-eye movement periods (SOREMPs) as rapid eye movement (REM) latencies in the course of OCD [24]. Additionally, with the aim to detect further potential clinical factors still accounting for treatment refractoriness, a systematic assessment of temperament and soft bipolar spectrum signs, as well as evaluation of other clinical features potentially linked to treatment refractoriness (Table 1), including 'poor insight' OCD (PI-OCD), was performed on all patients at week 12 using the lifetime Temperament Evaluation of the Memphis, Pisa, Paris, and San Diego lifetime Autoquestionnaire (TEMPS-A) [25] and the Brown Assessment of Beliefs Scale (BABS) [26] which was also administered at baseline. The BABS is a seven-item questionnaire rating a number of dimensions that underlie delusional and non-delusional beliefs, developed to assess the degree of insight of illness, a clinical feature generally directly related to the propensity toward SRI response [27,28]. The TEMPS-A is a comprehensive questionnaire specifically developed to assess temperamental (for example, cyclothymic) or personality (for example, cluster A) traits that may account for lack of response to standard medications or even for worsening of the clinical picture on antidepressants (including increased risk for suicidal attempts) [29-31]. Finally, all the patients appeared steadily compliant toward medications and underwent weight monitoring as well liver function tests (LFTs) at baseline and at week 12 (all negative) in accordance with the recent guidance proposed by the European Agency of Medicines (EMEA) [32].

**Table 1:** Examples of clinical factors potentially linked to obsessive-compulsive disorder (OCD) treatment-refractory responses

Eventual OCD clinical features contributing to OCD refractory response toward SRI antidepressants	Assessment	Sample references by first author and year
Poor-insight feature, global severity of OCD	Clinical interview/BABS/Y-BOCS	Ravi Kishore 2004 [28]; Goodman 1989 [20]

Age at onset, familial history, duration of untreated illness and other sociodemographic features	Clinical interview/SCID-I demographics	First 1997 [18]; Fornaro 2009 [7]

Axis-I comorbidities (for example, MDD/other anxiety disorders)	Clinical interview/SCID-I/HAM-D/HAM-A	First 1997 [18]; Fornaro 2009 [7]; Hamilton 1960 [21]; Hamilton 1959 [22]

Temperamental/personality traits	Clinical interview/TEMPS-A	Hantouche 2003 [29]; Akiskal 2005 [25]

Medical comorbidities, including those affecting the cognitive status	Clinical interview/specific assessment	Fornaro 2009 [7]

Impaired circadian rhythms?	BRIAN	Giglio 2009^a ^[23]

^a^Further clinical features may predict treatment-refractory response to SRIs among OCD patients; while the chance of response tends to reduce with increase of total comorbidities and higher severity of illness, the eventual impact of some features as impairment of circadian rhythm still needs further assessment.

BABS = Brown Assessment of Beliefs Scale; BRIAN = Biological Rhythms Interview of Assessment in Neuropsychiatry; HAM-A/D = Hamilton Scale for Anxiety/Depression; MDD = major depressive disorder; SCID-I = Structured Clinical Interview for the Diagnostic and Statistical Manual of Mental Disorders, fourth edition (DSM-IV) Axis-I; SRI = serotonin reuptake inhibitor; TEMPS-A = Temperament Evaluation of the Memphis, Pisa, Paris, and San Diego lifetime Autoquestionnaire; Y-BOCS = Yale-Brown Obsessive Compulsive Scale.

At the end of the study, three out of six patients, in particular those reporting higher subjective sleep disturbances at baseline but not at the end point, showed a clinically significant OC improvement (Y-BOCS reduce by ≥35%). Interestingly, patients largely unchanged within the 3 month trial with 50 mg/day agomelatine (n = 3) did not report any relevant circadian rhythm impairment at baseline or end point, but were more suggestive for 'soft bipolar' features according to the TEMPS-A evaluation (two out of three had a cyclothymic temperament) and/or had poor insight features.

Concerning the side-effect profile, agomelatine 50 mg/day was a generally well tolerated therapy, although one patient exhibited weigh gain and another experienced, transient (spontaneously remitting) dizziness despite a prudent downtitration from the previous SRI medication.

## Case series

**Figure 1:**
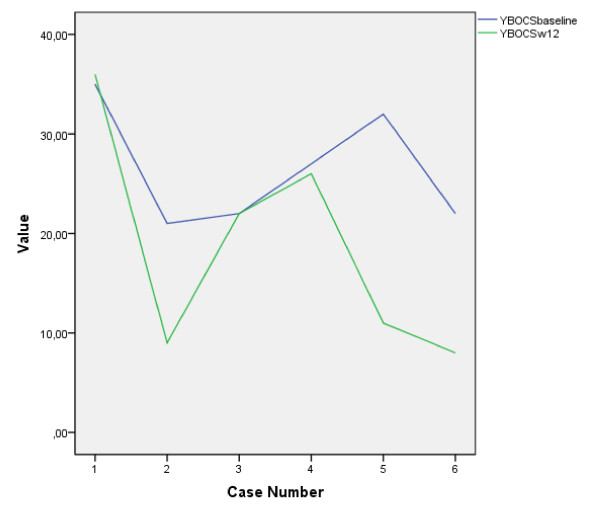
**Yale-Brown Obsessive Compulsive Scale (Y-BOCS) trends within 3 months of administration of agomelatine 50 mg/day in serotonin reuptake inhibitor (SRI)-refractory obsessive-compulsive disorders (OCDs)**. When remission occurred, it was substantial. In contrast, those refractory to agomelatine in the trial were largely unchanged within a 3 month follow-up.

The first patient was a single 35-year-old worker, who first started to develop OC symptoms at the age of 23. A year after being left by his girlfriend, he showed a sudden worsening of the disorder. (Clinical and demographical characteristics of all patients are summarized in Table 2 and Figure 1.) He was obsessively worried about the chance his ex-fiancé would get engaged to someone else and concerned about his subsequent inability to work. Compulsive shopping for audio CDs and sporadic cocaine use were then associated with a progressive social withdrawal (fulfilling DSM-IV criteria for social anxiety disorder (SAD)) and continuous ruminative/dubitative thoughts. The patient had already reported a history of partial/no response to SRIs and resistant MDD prior receiving 60 mg/day of fluoxetine for 6 months without exhibiting any further substantial benefit (Y-BOCS score = 35). After a 3 week wash-out period from fluoxetine, depressive and anxious symptoms were assessed and quantified with a HAM-D21 of 27 (severe), HAM-A of 44 (very severe) and BRIAN scale highlighting almost steady 'social' and 'predominant rhythm' impairments but not relevant subjective disruption of sleep rhythm within the whole agomelatine trial. According to a BABS score of 12 and a score of 2 for the 'conviction' item in particular, the patient maintained a substantial insight of illness [33]. Agomelatine was then started at 50 mg/day but all the monthly assessment Y-BOCS measurements were largely stable, with a week 12 total score of 36 (slight increase, largely not modified) and a week 12 BABS score of 11. Weight gain (+4 kg in 3 months; baseline BMI = 32; end point BMI = 34: second degree obesity) was the only relevant side effect. Interestingly, upon suggestion of a plausible cyclothymic temperament according to the clinical impression and administration of TEMPS-A at week 12, the patient was then switched to lithium carbonate 900 mg/day (lithium serum levels = 0.74 mEq/L) plus quetiapine 300 mg/day, showing a slight clinical reduction of OC symptom load just after a further month of antidepressant-free therapy and a remarkable, unexpected reduction of body weight by 3 kg.

**Table 2:** Clinical and demographic characteristics of SRI-refractory OCD patients treated with agomelatine 50 mg/day for a minimum of 3 months

	Patient 1	Patient 2	Patient 3	Patient 4	Patient 5	Patient 6
Age	35	44	66	53	25	33

Age at onset	23	36	57	20	15	24

Gender	M	M	F	F	M	F

DSM-IV Axis-I comorbidities	Resistant MDD, social anxiety disorder	Impulse-control disorder, substance abuse disorder	None	Bulimia nervosa, panic disorder	None	Panic disorder

First-degree relatives family history	Bipolar disorder II (brother)	ADHD (son)	None	None	None	None

Side effects	Weight gain (BMI 32 to 34 within 3 months)	None	None	None	None	Dizziness (initial, spontaneously remitting)

Last failed SRI trial	Fluoxetine 60 mg/day	Sertraline 200 mg/day	Fluoxetine 60 mg/day	Amitriptyline 300 mg/day	Fluvoxamine 250 mg/day	Duloxetine 120 mg/day + CBT

Main clinical features/OC symptoms	Doubt/compulsive shopping	Belching compulsion	Religious/touching	Hoarding/insertion of thought and intrusive images with sexual contents/checking	Checking/repeating	Fear of losing control toward food (in absence of BED)

Y-BOCS total score (baseline to week 12)	Not modified	Remission: 21 to 9	Not modified	Not modified	Remission: 32 to 11	Remission: 22 to 8

Actual, subjective circadian rhythms impairment (BRIAN)	Any relevant disruptions	Reported stable insomnia	Any relevant disruptions	Any relevant disruptions	Reported daily hypersomnia and nocturnal awakening	Any relevant disruptions

Lifetime bipolar spectrum signs (TEMPS-A at week 12)	Cyclothymic temperament	Anxious temperament	Any specific	Cyclothymic temperament	Any specific	Any specific (but presence of PD)

Poor insight feature (BABS, baseline-week 12)	No (stable)	Yes→No	No (stable)	No (stable)	No (stable)	No (stable)

Patient 6 received CBT within the 3 month trial with agomelatine; psychotherapy had already been established at least 6 months earlier.

ADHD = attention deficit hyperactivity disorder; BABS = Brown Assessment of Beliefs Scale; BED = Binge Eating Disorder; BMI = body mass index; BRIAN = Biological Rhythms Interview of Assessment in Neuropsychiatry; CBT = cognitive behavioral therapy; DSM-IV = Diagnostic and Statistical Manual of Mental Disorders, fourth edition; MDD = major depressive disorder; SRI = serotonin reuptake inhibitor; OC = obsessive compulsive; PD, panic disorder; TEMPS-A = Temperament Evaluation of the Memphis, Pisa, Paris, and San Diego lifetime Autoquestionnaire; Y-BOCS = Yale-Brown Obsessive Compulsive Scale.

The second patient was a 44-year-old trader who received his first diagnoses of OCD, impulse control and substance abuse disorders at the age of 36. His OC symptoms were essentially represented by repetitive belching compulsions and fear about losing self-control in the presence of other people, especially his customers, with consequent impact on his ability to work. The typical OCD egodystonic characteristic was almost lost over the years, leading to trials of multiple SRI prescriptions (including clomipramine 300 mg/day and other tricyclic antidepressants (TCAs)) augmented with the typical antipsychotic pimozide (4 mg/day) for the 'poor insight' feature (confirmed by a baseline BABS total score of 28 with 'conviction' item score = 4, the maximum possible score, inversely related to insight of illness). His last SRI prescription was sertraline 200 mg/day corresponding to a Y-BOCS score of 21 (HAM-D21 = 15; HAM-A = 16; BRIAN 'seldom' = acceptable in most of the domains but with severe, stable subjective initial insomnia already unresponsive to benzodiazepines (BDZs) and antihistamine drugs) prior to a 2 week wash-out period and subsequent therapy with agomelatine 50 mg/day. Monthly scheduled Y-BOCS score measurements indicated a progressive and fast, substantial reduction (Y-BOCS at week 4 = 16; week 8 = 11; week 12 = 9; week 12 BABS of 10 and subjective improvement sleep quality according to the BRIAN) without relevant side effects. Actual pharmacological regimen, including 4 mg/day pimozide and the anticholinergic drug biperidone 4 mg/day augmentation (added to prevent extrapyramidal symptoms), was confirmed in the presence of a TEMPS-A-demonstrated anxious temperament.

The third patient was a married, 66-year-old retired woman whose OC symptoms consisted of religious obsessions, continuous rituals regarding touching holy pictures of saints in her pocket and continuative fear of cursing against God. Both cognitive status and insight of illness (as measured by a BABS total score of 11) were maintained without any significant personal or familial medical or psychiatric anamnesis. The patient showed her first symptoms at the age of 57 when she experienced the loss of her only daughter, a nun. When the patient retired from her occupation (teacher) at the age of 60 she progressively experienced a worsening of her symptomatology, which became more time consuming until she finally sought medical care. She underwent different 'adequate' SRI trials (both with TCAs and SSRIs) prior to being treated with fluoxetine 60 mg/day. Before being switched to agomelatine 50 mg/day (after the 3 week wash-out period for fluoxetine), her symptomatology was quantified as Y-BOCS = 22, HAM-D21 = 23 and HAM-A = 25, while the BRIAN scale showed a predominant 'activity' pattern impairment ('sometimes') but not a remarkable impairment of sleep pattern. None of the subsequent observations showed modification in the reference scores apart from HAM-A, which was reduced by 2 points (HAM-A = 23) after the 12 week follow-up assessment; her week 12 BABS score was 10 and Y-BOCS = 22 at baseline. Analogously, administration of TEMPS-A did not show any relevant temperamental feature.

The fourth patient was a 53-year-old painter who developed her first OC symptoms at the age of 20. The patient also reported a history of bulimia nervosa (BN) diagnosed at the age of 24 (apparently spontaneously recovered after 8 years) and a lifetime history of panic disorder (PD) diagnosed by the age of 21. Her OC symptoms, mainly consisting of hoarding, insertion of thought (sexual images when painting fruits or other objects which shape could resemble her the male genitals) and checking rituals were very disabling and she said they might have eventually lead her to 'commit suicide to stop her sufferance. The patient had a firm insight of illness (baseline BABS total score = 9). The patient was treated with CBT from the age of 29, leading to repetitive failure and a final cessation of any psychotherapeutic support. She started her pharmacotherapy with amitriptyline 300 mg/day at the age of 44 exhibiting a transient response. Although reporting an episodic course of anxious illness (instead of a chronic waxing course) and BN and PD predictive factors, she had no personal or familial history for bipolar disorders (BD) but had a TEMPS-A result suggestive for cyclothymic temperament, despite absence of manic switch history even at considerable TCA doses without mood stabilizers. Before being switched to agomelatine 50 mg/day (after 2 week wash-out period for amitriptyline), her symptomatology was quantified by Y-BOCS = 27, HAM-D21 = 23 and HAM-A = 17 scores, while the BRIAN scale result showed a predominant 'social' pattern impairment ('often' = most of the time) but not sleep core disruptions. Over the whole 3 month follow-up, agomelatine was not associated with any side effects, including (hypo)mania, but was not related to any clinical improvement either (at week 12, Y-BOCS = 26 and BABS = 9).

The patient decided to return to her psychotherapist, disregarding the advice of waiting longer for a potential onset of action of the pharmacological treatment, switching to an atypical antipsychotic, or the suggestion of a combined approach.

The fifth patient was a 25-year-old student who experienced his first OC symptoms at the age of 15, predominantly consisting in checking behavior (door locks, gas knobs on the cooker and so on) and repeating, schizotypal-like (baseline BABS = 13) rituals (for example, mentally counting pairs of numbers in a 10 min period every 3 h). Considering the age of the subject and the early onset of the symptoms, OCD appeared severe, as confirmed by a Y-BOCS total score of 32 even after an 8 month trial with fluvoxamine 250 mg/day, with relevant social withdrawal and (law) college course abandonment associated with daily hypersomnia and nocturnal awakening. No familial or personal comorbidities were shown. At that point, his HAM-D21 total score was 25, HAM-A = 39 and BRIAN results showed almost constant 'chronotype' and 'sleep' disruptions. After a 2 week SSRI wash-out period, the patient started a 50 mg/day agomelatine trial, showing a progressive, considerable remission (Y-BOCS at week 4 = 19, Y-BOCS at week 8 = 13, Y-BOCS at week 12 = 11 and week 12 BABS = 8). Almost all the OC symptoms appeared to be in remission and his circadian rhythms recovered, suggesting continuation of the same therapy in the absence of any relevant TEMPS-A temperamental cluster. Finally, no side effects were reported during the treatment.

The sixth and final patient was 33-year-old waitress who developed her first OC symptoms at the age of 24. Her obsessions were mainly related to fear of lack of control toward food. Curiously, she had no personal or familial history for any eating disorder (ED), including binge eating disorder (BED) or night eating, or any dysmorphophobic bodily perceptions, but had a personal history of PD with episodic attacks since the age of 18. She had a history of repetitive failure on antidepressant trials, with the last one being the SNRI duloxetine 120 mg/day and the BDZ clonazepam 4 mg/day for 3 months plus CBT for 6 months. At baseline, her Y-BOCS total score was 22 and BABS = 9 with HAM-D21 = 22 and HAM-A = 35. BRIAN administration did not show any relevant impairment. After a 2 week SNRI and BDZ wash-out period, the patient started a 50 mg/day agomelatine trial, showing a progressive, consistent remission (Y-BOCS at week 4 = 19; Y-BOCS at week 8 = 14; Y-BOCS at week 12 = 8 and week 12 BABS = 8). TEMPS-A temperamental evaluation did not show any relevant cluster. Finally, the patient accepted continuing the therapy; no side effects were reported during the treatment but modest, transient, self-remitting dizziness despite a progressive downtitration of venlafaxine prior to the agomelatine switch. She experienced no panic attacks during the whole period she was taking agomelatine.

## Discussion

In the present case series the efficacy of a 12 week trial of agomelatine (50 mg/day) has been assessed in six patients refractory to previous treatments with SRIs, with and without other psychiatric comorbidities. At the end of the treatment, three out of six patients showed a clinical improvement with a symptom reduction ≥35% over the pretreatment Y-BOCS scores, which is considered a significant treatment response in current clinical trials [6].

Although recently introduced, agomelatine appears to be an effective MDD treatment [34], but to date there has been no investigation with regard to its potential role in the management of OCD.

However, the 5-HT2C modulation and subsequent norepinephrine and dopamine firing disinhibition (NDDI action) at the prefrontal cortex, with reduction of stress-induced increase of glutamate via synergy with melatonergic and 5-HT2C receptor-dependent pathways [35], as well as the influence of MT1 and MT2 agonism (which in case of agomelatine is a mechanism predominant toward the 5-HT2C blockade) on circadian rhythms (impaired in some OCD cases), might suggest a potential role of agomelatine in the management of anxiety disorders, including OCD, as already analogously proposed for SAD [36] and PD [17].

Additionally, the efficacy of SRIs, including the widely prescribed SSRIs, for mood and anxiety disorders has been questioned [37], prompting the exploration of other classes of drugs including NDDIs with proof of direct 5-HT2A stimulating action, such as trazodone, mirtazapine and agomelatine.

With regard to the dose of agomelatine for OCD, there is no actual evidence to suggest starting at 50 mg/day or its initial introduction at 25 mg/day, although agomelatine has been reported to be a generally well tolerated treatment without the need for a specific uptitration [38]. Additionally, OCD is a generally harder to treat condition compared to MDD as is shown by the fact that acceptable response rates (Y-BOCS ≤35%) are lower than the corresponding ones adopted for MDD remission (HAM-D17 ≤50%), and that the clinical practice often suggests the use of higher doses of (serotonergic) drugs for OCD in comparison to MDD (the approved range for agomelatine is 25-50 mg/day).

While the determination of appropriate dosage of agomelatine in course of SRI-refractory OCD is beyond the scope and possibilities of this report, it is likely that a 'dose bias' might further influence the validity of these very preliminary results, already conditioned by methodological issues, a tiny, heterogeneous sample size and short follow-up. Additionally, a number of issues, including OCD subtype (for example, 'hoarding') and comorbidities (for example, severe mood disorders and other anxiety disorders) and bipolar-related temperaments, could also account for the therapeutic outcome.

Nonetheless, it remains unclear if and how the presence of impaired circadian rhythms such as sleep disruption (or even other features as seasonal course of illness) might influence the therapeutic response of OCD especially considering the lack of objective neurophysiological monitoring. In fact, in this report agomelatine appeared effective for those SRI-refractory OCD patients who also subjectively reported impaired sleep (none of them had TEMPS-A findings, but there was 'anxious temperament' in one case (patient 2), and the presence of a cyclothymic temperament in the absence of impaired circadian rhythm (patients 1 and 4)) was associated to refractoriness to agomelatine within 3 months of 50 mg/day therapy. This appears to be consistent with evidence in the current literature pointing out a lack of response to antidepressants (usually to SRIs) among those (OCD and/or MDD) patients with 'soft bipolar' characteristics, including 'cyclothymic sensitiveness' and 'interpersonal sensitivity' (inability to accept refusal from the ex-fiancé) or 'sensation seeking behavior' (cocaine use) [39]. Other clinical features such as behavioral addictions, substance abuse, impulse control disorders, bulimia, comorbidity with PD (for example, patient 6), sexual/religious obsessions, late onset of OC symptoms after grief (for example, patient 3), concomitant depression (high scores on the HAM-D) might also be accounted for by potential 'hidden bipolar' signs or as generic factors responsible to antidepressant refractoriness among OCD patients.

As consequence, even the consideration of a SRI switch in favor of agomelatine among OCD refractory patients should prompt for attentive evaluation of subthreshold bipolarity and eventually suggest an early administration of lifetime screening instruments such as TEMPS-A along with a more accurate clinical interview or a prompt consideration of mood stabilizers such as lithium, antiepileptic drugs and atypical antipsychotics[39]. Nonetheless, further investigations should assess whether agomelatine might be less prone to induce (hypo)mania in comparison to standard antidepressants, as indirectly suggested by criteria '2a' and '2b' for 'rhythm regulator' recently proposed by Fountoulakis [40].

Among those with impaired circadian rhythms prior switching to agomelatine (all were remitters at week 12), it should be noted that agomelatine has already been associated with early recovery of sleep disruption when associated with MDD [32]. Circadian rhythm restoration usually precedes the 'antidepressant' response in comparison to other classes of antidepressants, which in turn might be associated with circadian rhythm disruption [32]. Nonetheless, considering the absence of objective recording and the intrinsic limitations of current rating instruments [32] it is difficult to discriminate whether the subjective amelioration of circadian rhythm should also be perceived as an improvement of affective and/or anxious symptoms (this might be the case of patients 2, 5 and 6), as this remains a major task to be assessed by further controlled investigations.

Other concerns regard the augmentation strategy adopted for patient 2: in fact, it is difficult to understand if and how the presence of an incisive D2 blocker, first generation antipsychotic (pimozide) could have favored (or eventually induced *per se*) the response during agomelatine treatment in the presence of PI-OCD.

Gender and age could also influence the OCD response to SRIs [7] and this should be valid also for agomelatine, as potentially occurred in patient 3. Similarly, life stress may lead to mood and/or anxiety disorders (including OCD) by causing disruptions in individuals' social routines and, in turn, their biological rhythms according to the 'social zeitgeber' theory [41], eventually representing OCD features more favorably responding to MT1 and MT2 agonists such as agomelatine (patient 5) [32].

Indeed, many other factors may influence the clinical presentation of OCD and its putative response to agomelatine. Among others, the role of concomitant psychotherapy (CBT) might be better investigated. However, in this case series it does not appear to be a confounding factor influencing the outcome of agomelatine treatment, since patient 6 receiving psychotherapy had already been treated with CBT for at least 6 months in the course of SRI treatment(s).

With regard to the side effects experienced during agomelatine treatment, weight gain (patient 1) appears to be an unexpected occurrence. In fact, agomelatine is considered generally safe regarding weight gain, especially versus SRIs [32], which the patient had already been taking for about 4 years prior to the agomelatine switch without experiencing significant weight gain. Also, patient 1 showed a slight reduction of body weight just after 1 month of therapy with lithium carbonate and quetiapine, an atypical antipsychotic drug which also blocks the 5-HT2C receptors via its principal active metabolite (norquetiapine) and also acts as an antihistamine and 5-HT2A blocker [32]. However, the effective role of 5-HT2C blockade on weight gain is still under debate as it may also observed that the use of 5-HT2C stimulants has been proposed for obesity, which on turn appears to occur more frequently among individuals reporting sleep disturbances [42]. Nonetheless, in this case the effect of mood stabilization appeared to be the most effective antiobesity therapeutic intervetion.

Concerning dizziness, experienced by patient 6, this appears to be a known issue potentially occurring with the beginning of an agomelatine trial [32]. Nonetheless, when it occurs, it is supposed to be due to a 'flu-like' syndrome (for example, dizziness, general malaise, fever, diarrhea) associated with sudden serotonergic (especially SSRI) withdrawal and consequent rebound syndrome, hypothetically due to abrupt interruption of 5-HT2A postsynaptic stimulation (in the presence of SRI-upregulated receptors) as it may happen even with agomelatine, which is proof of (direct) 5-HT2A stimulating action malaise [43]. However, in case 6, the patient was prudently downtitrated from the SNRI venlafaxine and the BDZ clonazepam so that dizziness appeared as an unexpected occurrence, which in this case was speculated to be due to a peculiar cerebellar sensitivity of MT1 and MT2 neurons. Remarkably, the degree of severity of transient, self-remitting dizziness did not preclude continuation of agomelatine.

Finally, despite a number a methodological and clinical constraints and the very preliminary nature of the results, further controlled studies are warranted to assess the efficacy of agomelatine in course of OCD, with more accurate methodological investigation od the eventual role of circadian rhythm imbalance, even in the course of drug-naïve cases with no other relevant comorbidities.

## Consent

Written informed consent was obtained from the patients for publication of this article.

## Competing interests

The author declare that he has no competing interests.
